# Optical Coherence Tomography Angiography Assessment of the Peripapillary Vessel Density and Structure in Patients with Nonarteritic Anterior Ischemic Optic Neuropathy: A Meta-Analysis

**DOI:** 10.1155/2020/1359120

**Published:** 2020-10-29

**Authors:** Ling Ling, Kaibao Ji, Liping Xie, Feifei Gao, Qinglin Zhang, Yiqiao Xing, Wentian Zhou

**Affiliations:** ^1^Affiliated Eye Hospital of Nanchang University, Nanchang Jiangxi, China; ^2^Department of Ophthalmology, Renmin Hospital of Wuhan University, Wuhan Hubei, China; ^3^Department of Ophthalmology, Jiangxi Province Cancer Hospital, Nanchang, Jiangxi, China; ^4^Department of Ophthalmology, Huangshi Central Hospital, Affiliated Hospital of Hubei Polytechnic University, Edong Healthcare Group, Huangshi, China

## Abstract

**Background:**

This meta-analysis is aimed at assessing the peripapillary vessel density (VD) and structural outcomes using optical coherence tomography angiography (OCTA) in patients with nonarteritic anterior ischemic optic neuropathy (NAION).

**Methods:**

A comprehensive search of PubMed, Embase, Cochrane Library, and Web of Science databases for literature comparing VD and structural outcomes in patients with NAION and controls was performed. Mean differences (MDs) and its 95% confidence interval (CI) were calculated for continuous estimates. Review Manager (V5.30) was used for analysis.

**Results:**

Fourteen published studies met the requirement. The radial peripapillary capillary (RPC) whole enface VD measured by OCTA was significantly lower in patients with NAION compared to that of the controls (MD = −10.51, *P* < 0.00001). The RPC inside disc VD was significantly decreased in the NAION group than that in the control group (MD = −8.47, *P* < 0.00001). For RPC peripapillary VD, there was a statistically significant difference between patients with NAION and the controls (MD = −12.48, *P* < 0.00001). The peripapillary retinal nerve fibre layer (p-RNFL) thickness was significantly lower in patients with NAION in comparison to the controls (MD = −22.18, *P* = 0.004). The ganglion cell complex (GCC) thickness in the macular zone of NAION patients was remarkably reduced compared to that in the controls (MD = −17.18, *P* = 0.0002).

**Conclusions:**

The findings suggested that the peripapillary VD and RNFL thickness were attenuated, and the macular GCC thickness was reduced in patients with NAION. OCTA, in the future, may facilitate the diagnosis and monitoring of patients with NAION.

## 1. Introduction

Nonarteritic anterior ischemic optic neuropathy (NAION) is a visually threatening optic neuropathy characterised by sudden and rapidly painless vision loss, altitudinal visual field defect, and optic disc oedema [1]. NAION is estimated to occur in 2.3-10.3 per 100,000 individuals in the middle-aged and elderly population [2, 3]. Although the underlying mechanism of NAION remains unknown, the available evidence reveals that it may be associated with perfusion deficiency of the optic nerve head (ONH) microcirculation that is predominantly supplied by the short posterior ciliary arteries [4–6]. The probable risk factors that increase the onset of NAION include hypertension, hypercholesterolemia, diabetes mellitus, nocturnal hypotension, and obstructive sleep apnoea [7–10].

Currently, several imaging modalities have been employed to detect optic disc microvasculature in NAION [11]. Among these imaging techniques, fundus fluorescein angiography (FFA) is currently the standard procedure to recognize the impaired ONH blood flow in NAION [12]. However, FFA is an invasive procedure and does not quantitatively assess optic disc vascular structures at different layers [13]. As for laser Doppler velocimetry, delayed ONH blood flow has been found in patients with NAION; however, it cannot provide quantitative assessment of the vasculature in the ONH [14]. Optical coherence tomography angiography (OCTA) is a new, noninvasive imaging technology that can quantify the blood flow within the retina and choroid without needing fluorescein dye injection [15]. Several studies conducted by OCTA have demonstrated decreased vessel densities in the ONH and radial peripapillary capillary (RPC) in the eyes with NAION [16–18]. Prior studies also revealed that the reduced vessel density of the peripapillary retina was significantly correlated with the peripapillary retinal nerve fibre layer (p-RNFL) thickness and visual field mean defect [19, 20]. However, to our knowledge, there has been no meta-analysis comprehensively investigating the peripapillary vessel density and structural features related to patients with NAION.

Therefore, the current meta-analysis was carried out to address this issue and provide robust evidence for ophthalmologists to properly manage patients with NAION.

## 2. Materials and Methods

### 2.1. Search Strategy

This meta-analysis was performed following the Preferred Reporting Items for Systematic reviews and Meta-Analyses (PRISMA) statement [21], and no ethical approval was needed. Two independent investigators comprehensively searched the electronic databases including PubMed, Embase, Cochrane Library, and Web of Science from inception through 31 May 2020 to identify relevant literature. To achieve maximum numbers of articles, the following search terms were used: ((((OCTA) OR (OCT angiography)) OR (optical coherence tomographic angiography)) OR (optical coherence tomography angiography)) AND (((Non-arteritic Anterior Ischemic Optic Neuropathy) OR (non-arteritic anterior ischemic optic neuropathy)) OR (NAION)). English-language articles were regarded as eligible. All discrepancies were resolved by deliberation with each other or discussion with the third author.

### 2.2. Study Selection

Studies were included in this meta-analysis if they met the following criteria: (1) they are original articles; (2) they are studies comparing patients with NAION (including acute and nonacute (equivalent to chronic) NAION) with healthy controls; (3) OCTA data were provided as mean ± standard deviation (SD); and (4) primary outcomes included radial peripapillary capillary (RPC) whole enface vessel density (VD), RPC inside disc VD, RPC peripapillary VD, p-RNFL thickness, and macular ganglion cell complex (GCC) thickness.

The exclusion criteria were as follows: (1) case series, abstracts, posters, animal studies, reviews, comments, and meta-analyses; (2) study objective did not meet the inclusion criteria; (3) duplicate publication from the same study; (4) study outcomes could not be extracted; and (5) study results were unclearly reported.

### 2.3. Data Extraction and Quality Assessment

Two independent authors (Ling Ling and Kaibao Ji) independently extracted the data from the included studies, and disagreements were resolved by discussion with the third author. The following data were collected from the included articles: first author, location, publication year, study design, sample size, mean age, sex ratio, type of OCTA, primary outcomes, and quality of a study. The quality assessment of the included studies was performed according to the Newcastle-Ottawa scale, which provided a score range of 0 to 9 points, with a higher score (≥5) indicating better quality [22].

### 2.4. Statistical Analysis

The Review Manager Software Version 5.30 (Cochrane Collaboration, Oxford, UK) was used for analysis. Mean differences (MDs) and its 95% confidence interval (CI) were calculated for continuous estimates. Heterogeneity among studies was performed using the chi-square statistic test and *I*^2^ statistic test. *I*^2^ values of 25%, 50%, and 75% represented mild, moderate, and high heterogeneity, respectively. A fixed-effect model was employed when no significant heterogeneity existed among studies; otherwise, the random-effect model was used. Potential publication bias was assessed by the funnel plot. *P* < 0.05 was considered a significant difference.

## 3. Results

### 3.1. Literature Search Results

**Figure 1:**
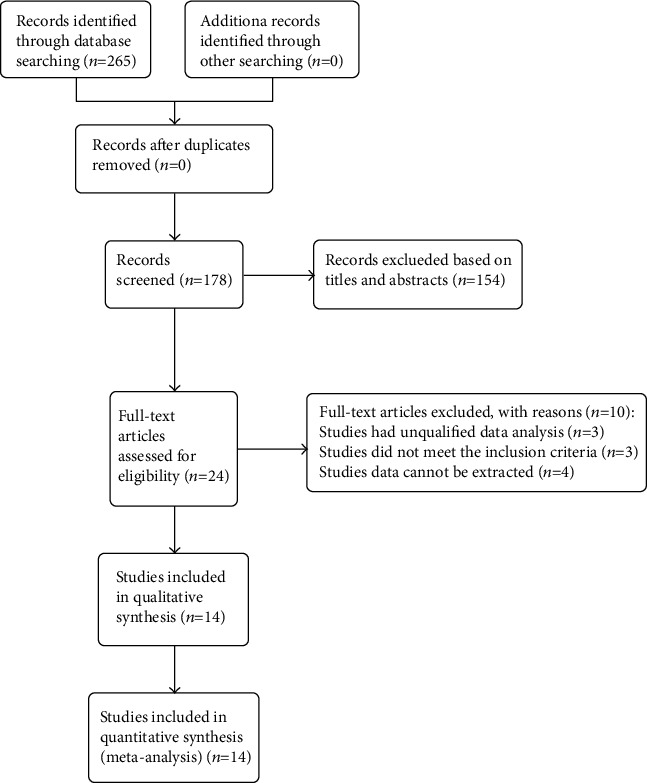
The selection process of literature retrieval and screening.

The selection process of literature retrieval and screening is presented in Figure 1. A total of 265 potential articles were initially searched from the databases (PubMed: 110, Web of Science: 50, Embase: 105, and Cochrane Library: 0), of which 87 duplicated articles were excluded. In addition, 154 articles were excluded after reviewing the titles and abstracts. The residual 24 articles were in detail screened for the full text; three studies had unqualified data, three did not meet the inclusion criteria, and for the other four studies, data could not be extracted. Finally, fourteen articles [19, 20, 23–34], with a total of 783 eyes (313 in the NAION group and 470 in the control group), were included in the meta-analysis.

The basic characteristics of the included articles are summarized in Table 1, and the quality assessment results of the included studies are reported in Table 2.

### 3.2. Main Results

#### 3.2.1. Peripapillary Vessel Density Analysis in Patients with NAION and the Controls

**Figure 2:**
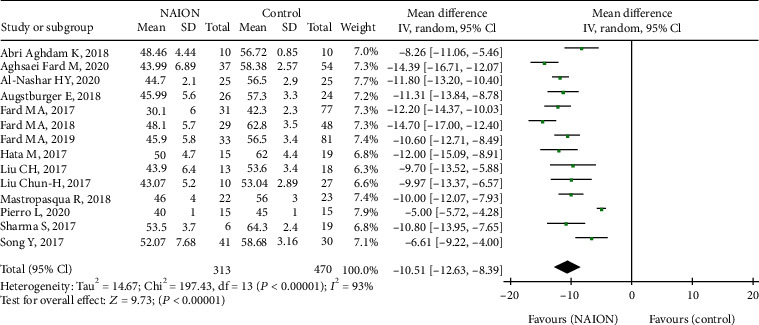
Forest plot showing RPC whole enface vessel density in NAION groups and control groups. RPC: radial peripapillary capillary.

**Figure 3:**
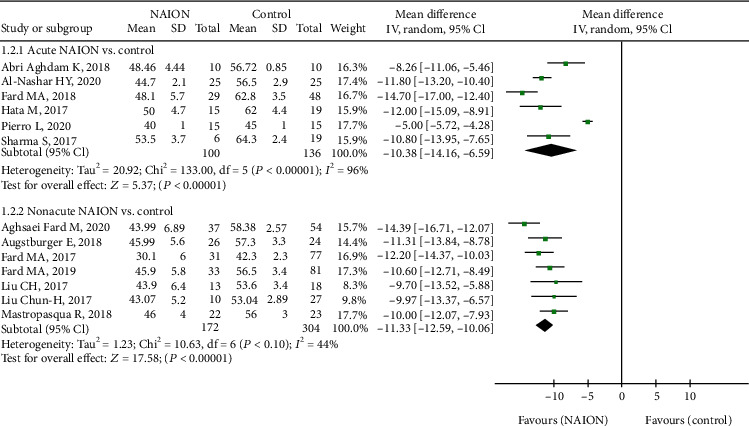
Forest plot for RPC whole enface vessel density between two subgroup analyses. RPC: radial peripapillary capillary.

All fourteen studies, including 783 eyes (313 in the NAION group and 470 in the control group), reported on the RPC whole enface vessel density. The pooled mean difference (MD) for RPC whole enface vessel density (VD) between the NAION and control groups was -10.51 (95% CI: -12.63 to -8.39, *P* < 0.00001, Figure 2), with significant heterogeneity across studies (chi^2^ = 197.43, *P* < 0.00001, *I*^2^ = 93%, Figure 2), showing that the RPC whole enface VD was lower in the NAION group. The subgroup analyses were also performed in this group. The pooled results indicated that the RPC whole enface VD was significantly lower in the acute NAION and nonacute NAION groups than that in the control groups (MD = −10.38, 95% CI: -14.16 to -6.59, *P* < 0.00001; MD = −11.33, 95% CI: -12.59 to -10.06, *P* < 0.00001, respectively, Figure 3), but there was substantial heterogeneity among the studies in the acute NAION subgroup (chi^2^ = 133.0, *P* < 0.00001, *I*^2^ = 96%, Figure 3).

**Figure 4:**
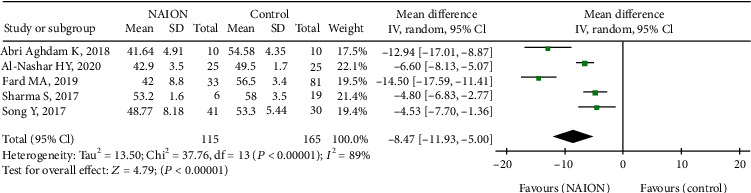
Forest plot comparing RPC inside disc vessel density in NAION patients and controls. RPC: radial peripapillary capillary.

**Figure 5:**
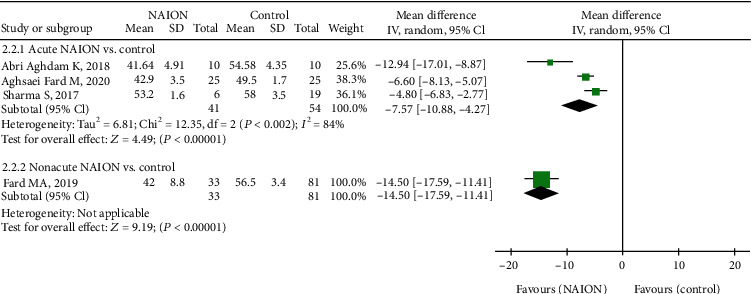
Forest plot for RPC inside disc vessel density in two subgroup analyses. RPC: radial peripapillary capillary.

In addition, five studies, including 280 eyes (115 in the NAION group and 165 in the control group), reported on the RPC inside disc VD of their participants. The summary MD in the RPC inside disc VD between these two groups was -8.74 (95% CI: -11.93 to -5.00, *P* < 0.00001, Figure 4), revealing that RPC inside disc VD was lower in patients with NAION, but a high heterogeneity was established among the studies for this outcome (chi^2^ = 37.76, *P* < 0.00001, *I*^2^ = 89%, Figure 4). The subgroup results also demonstrated that RPC inside disc VD was remarkably lower in the acute NAION group than that in the control group (MD = −7.57, 95% CI: -10.88 to -4.27, *P* < 0.00001, Figure 5), with substantial heterogeneity across studies (chi^2^ = 12.35, *P* = 0.002, *I*^2^ = 84%, Figure 5).

**Figure 6:**
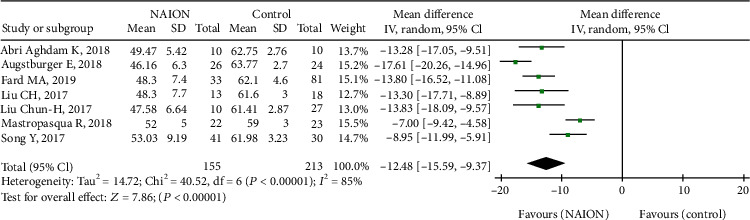
Forest plot indicating RPC peripapillary vessel density between patients with NAION and the controls. RPC: radial peripapillary capillary.

**Figure 7:**
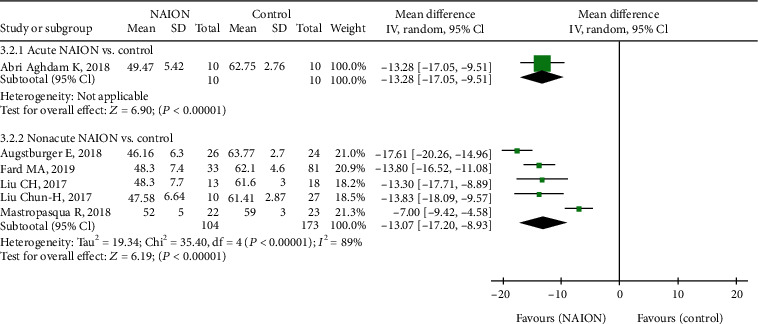
Forest plot for RPC peripapillary vessel density between two subgroup analyses. RPC: radial peripapillary capillary.

Furthermore, seven studies, including 368 eyes (155 in the NAION group and 213 in the control group), calculated the RPC peripapillary VD between the groups. The difference was significant between the two groups (MD = −12.48, 95% CI: -15.59 to -9.37, *P* < 0.00001, Figure 6), but there was high heterogeneity among the studies of this outcome (chi^2^ = 40.52, *P* = 0.002, *I*^2^ = 85%, Figure 6). The summary MD in the subgroup analysis was significantly lower in the nonacute NAION subjects than that in the control subjects (MD = −13.07, 95% CI: -17.20 to -8.93, *P* < 0.00001, Figure 7), but there was substantial heterogeneity found among the studies (chi^2^ = 35.40, *P* < 0.00001, *I*^2^ = 89%, Figure 7).

#### 3.2.2. Peripapillary Structure Analysis in Patients with NAION and the Controls

**Figure 8:**
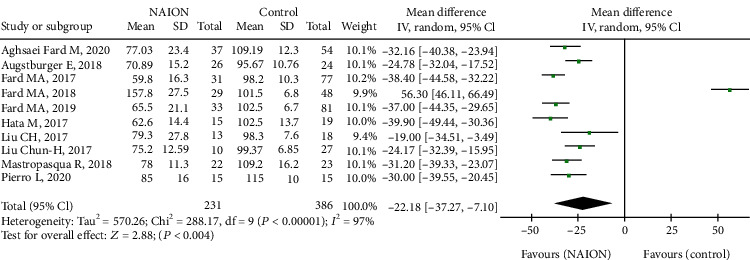
Forest plot of p-RNFL thickness in patients with NAION and the controls. p-RNFL: peripapillary retinal nerve fibre layer.

**Figure 9:**
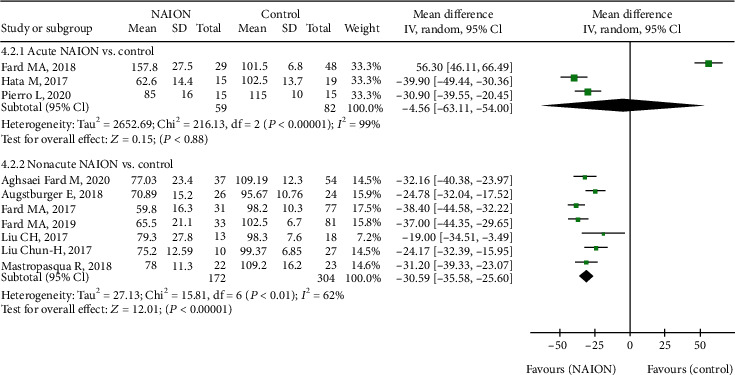
Forest plot for p-RNFL thickness in two subgroup analyses. p-RNFL: peripapillary retinal nerve fibre layer.

In terms of peripapillary structure, we analysed the p-RNFL thickness, showing that the pooled MD in the NAION group was significantly decreased than that in the control group (MD = −22.18, 95% CI: -37.27 to -7.10, *P* = 0.004, Figure 8), with high heterogeneity (chi^2^ = 288.17, *P* < 0.00001, *I*^2^ = 97%, Figure 8). In subgroup analysis, the p-RNFL thickness of the acute NAION group was comparable to that of the control group (MD = −4.56, 95% CI: -63.11 to 54.00, *P* = 0.88, Figure 9), but patients with nonacute NAION had significantly thinner p-RNFL than the controls (MD = 30.59, 95% CI: -35.58 to -25.60, *P* < 0.00001, Figure 9), and the heterogeneity in both subgroups was high (chi^2^ = 216.13, *P* < 0.00001, *I*^2^ = 99%; chi^2^ = 15.81, *P* = 0.01, *I*^2^ = 62%, respectively, Figure 9).

**Figure 10:**
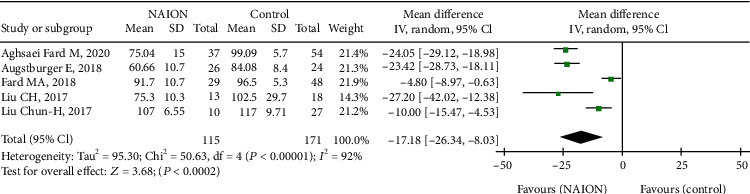
Forest plot of macular GCC thickness in NAION patients and controls. GCC: ganglion cell complex.

**Figure 11:**
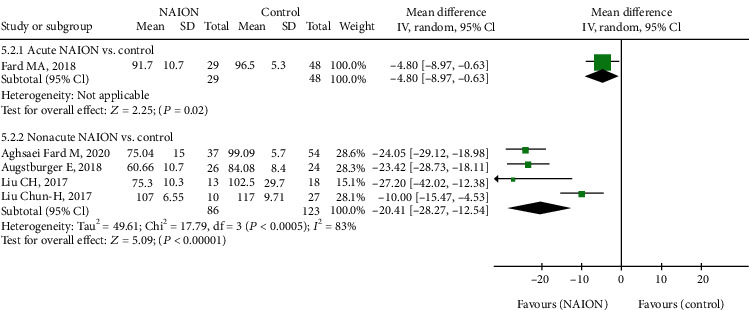
Forest plot for macular GCC thickness in two subgroup analyses. GCC: ganglion cell complex.

In addition, as to macular structure, we reported on macular GCC thickness between the NAION and control groups. There was significant thinning of GCC in the NAION group compared with the controls, with the MD of -17.18 (95% CI: -26.34 to -8.03, *P* = 0.0002, Figure 10) between the two groups and high heterogeneity among the studies (chi^2^ = 50.63, *P* < 0.00001, *I*^2^ = 92%, Figure 10). Then, the subgroup analysis indicated that GCC thickness of the nonacute NAION group was significantly lower than that of the control group (MD = −20.41, 95% CI: -28.27 to -12.54, *P* < 0.00001, Figure 11), and the heterogeneity was significant (chi^2^ = 17.79, *P* = 0.0005, *I*^2^ = 83%, Figure 11).

#### 3.2.3. Publication Bias

**Figure 12:**
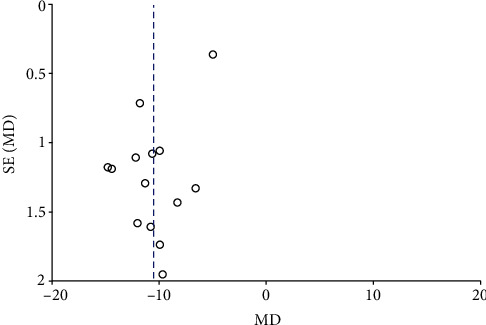
A funnel plot of RPC whole enface VD between patients with NAION and the controls showing no significant publication bias. RPC: radial peripapillary capillary; VD: vessel density.

**Figure 13:**
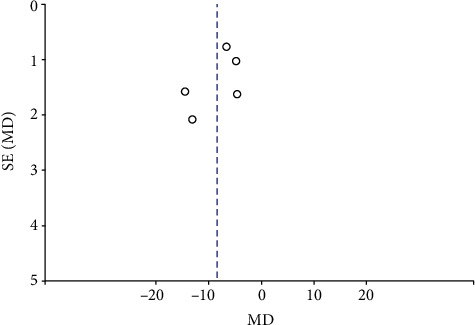
A funnel plot of RPC inside disc VD in NAION patients and controls indicating the absence of significant publication bias. RPC: radial peripapillary capillary; VD: vessel density.

**Figure 14:**
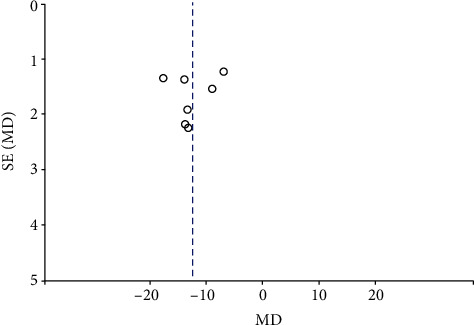
A funnel plot of RPC peripapillary VD in NAION patients and controls indicating the absence of significant publication bias. RPC: radial peripapillary capillary; VD: vessel density.

**Figure 15:**
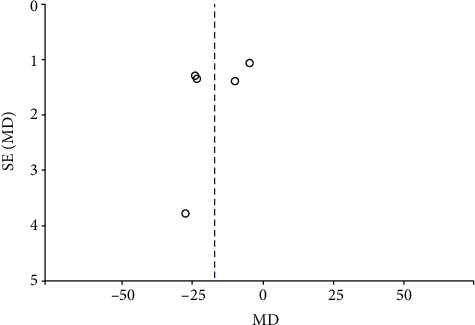
A funnel plot of macular GCC thickness in patients with NAION and the controls suggesting no significant publication bias. GCC: ganglion cell complex.

Funnel plots summarized the potential publication bias of RPC whole enface VD, RPC inside disc VD, RPC peripapillary VD, and macular GCC thickness among the studies. The results revealed that the distribution of studies was not an obvious asymmetry, indicating the absence of significant publication bias (Figures 12–15).

## 4. Discussion

To the best of our knowledge, this is the first meta-analysis to investigate the peripapillary microvascular and structural changes using OCTA in patients with NAION and control subjects. Fourteen eligible studies including 783 eyes were analysed in this meta-analysis. In the review, we pooled the mean RPC whole enface, RPC inside disc, and RPC peripapillary vessel densities of study participants, as well as their p-RNFL and macular GCC thicknesses. Our data figured out that there were significantly reduced peripapillary vessel density, p-RNFL thickness, and macular GCC thickness in patients with NAION compared to that of control subjects. And the funnel plots suggested no obvious publication bias.

Recently, some studies have used OCTA to assess the peripapillary microvascular in NAION eyes, suggesting reduced vessel densities of the RPC in the eyes with NAION, as well as reduced p-RNFL thickness [16–19]. Our current evidences reinforced the findings of the previous studies [35–37]. However, substantial heterogeneity was shown in our meta-analysis. Three studies contributed to the major heterogeneity of the RPC whole enface VD—Abri Aghdam et al. [23], Pierro et al. [32], and Song et al. [34]. The *I*^2^ measurement for this item significantly declined from 93% to 44% (chi^2^ = 17.97, *P* = 0.06), after the removal of the above three studies. In the acute-NAION subgroup, the heterogeneity also remarkably decreased from 96% to 46% (chi^2^ = 5.58, *P* = 0.13), when the two studies were excluded [23, 32]. Although these three studies were excluded from the analysis, the MDs for all were statistically significant. We also found that the other two studies [19, 23] contributed maximum heterogeneity to the RPC inside disc VD, revealing that heterogeneity considerably decreased from 89% to 23% (chi^2^ = 2.60, *P* = 0.27), when these two studies were excluded. The acute-NAION subgroup heterogeneity also subsequently decreased from 89% to 48% (chi^2^ = 1.93, *P* = 0.16), after the study was removed [23]. There was only one study that reported RPC inside disc VD in the nonacute NAION subgroup; therefore, no heterogeneity was observed. The MDs were still significant after the removal of these two studies—Fard et al. [19] and Pierro et al. [32], but the sample numbers were relatively small within the groups. As to RPC peripapillary VD, two studies contributed majorly to the heterogeneity of this analysis—Mastropasqua et al. [20] and Song et al. [34]. The *I*^2^ measurement for this parameter significantly declined from 85% to 34% (chi^2^ = 6.10, *P* = 0.19) after removing the above two studies, subsequently resulting in decreased heterogeneity from 89% to 43% (chi^2^ = 5.30, *P* = 0.15) in the nonacute NAION subgroup. No heterogeneity was found in the acute NAION subgroup, as merely one study reported the data. The MD estimates of this parameter were still significantly different after the two studies were excluded from analysis, suggesting results were robust.

Previous studies revealed that the peripapillary RNFL and GCC thicknesses decreased in the NAION eyes [38, 39]. In addition, reduced peripapillary VD was significantly correlated with p-RNFL thickness loss and visual field defect [20, 24, 40]. Here, we also reported decreased p-RNFL thickness and macular GCC thickness in patients with NAION compared with controls, but with significant heterogeneity. We observed that the mean p-RNFL thickness was the lowest in Fard et al. [27]; however, the mean p-RNFL thickness was the highest in Fard et al. [28]. This may contribute to the high heterogeneity of the studies. The apparent heterogeneity decreased from 97% (chi^2^ = 288.17, *P* < 0.00001) to 49% (chi^2^ = 13.71, *P* = 0.06) after excluding these two studies, and the pooled result was similar (*P* = 0.004; *P* < 0.00001, respectively). In subgroup analysis, the p-RNFL thickness in the acute NAION group was significantly reduced compared to that of the controls (*P* = 0.88; *P* < 0.00001, respectively) after removing Fard et al. [28], and the heterogeneity significantly decreased from 99% (chi^2^ = 216.13, *P* < 0.00001) to 52% (chi^2^ = 2.07, *P* = 0.15). Meanwhile, the heterogeneity in the nonacute NAION subgroup also decreased from 62% (chi^2^ = 15.81, *P* = 0.01) to 48% (chi^2^ = 9.57, *P* = 0.09) after removing Fard et al. [27], but the pooled result was similar (*P* < 0.00001; *P* < 0.00001, respectively) [27]. In addition, two other studies contributed significant heterogeneity to GCC thickness [28, 31]. Homogeneity was reached after removing these two studies together (chi^2^ = 0.22, *P* = 0.89, *I*^2^ = 0.0%). The homogeneity was achieved in the nonacute NAION subgroup analysis after excluding the study—Liu et al. [31].

All the above results suggested that the reduced peripapillary VD with NAION was unlikely to be due to the selection bias. We speculated some other factors, for instance, mean age of patients with NAION, percentage of male or female, sample size, geographical area, and ethnic differences, which may have contributed to the high heterogeneity in our analysis.

There are several potential limitations to be considered in our meta-analysis. Firstly, the sample size included in the study was comparatively small and the quality of the evidence was relatively low. Secondly, the pooled estimates should be interpreted carefully, as high heterogeneity existed in this meta-analysis. Thirdly, the source of heterogeneity could not be fully determined as a result of insufficient data to perform comprehensive metaregression. Finally, although we did not register the protocol of our study in the PROSPERO database, no corresponding protocols of this subject were found in the database. Future prospective and large-scale cohort studies or intervention trials should be carried out to validate our results.

In conclusion, our findings showed that the peripapillary vessel density and RNFL thickness were attenuated in patients with NAION when compared with controls. Furthermore, we revealed that the macular GCC thickness was also reduced in patients with NAION. OCTA, in the future, may become a useful technique in the diagnosis and monitoring of patients with NAION.

## Figures and Tables

**Table 1:** The basic characteristics of the included studies.

Study	Place	Mean age (years)	Study design	Number of eyes	Gender (M/F)	OCTA type	RPC scan size (mm)	Outcomes
Fard et al. [19]	Iran	55.8 ± 10.6 51.5 ± 19.5	Cross-sectional study	Cases: 33 Controls: 81	None	Optovue	4.5 × 4.5	RPC whole enface VD, RPC inside disc VD, RPC peripapillary VD, p-RNFL thickness
Mastropasqua et al. [20]	USA	68.1 ± 4.3 63.9 ± 7.0	Case-control study	Cases: 22 Controls: 23	9/13 12/11	Optovue	4.5 × 4.5	RPC whole enface VD, RPC peripapillary VD, p-RNFL thickness
Abri Aghdam et al. [23]	Iran	56.80 ± 6.81 27.90 ± 11.70	Prospective observational study	Cases: 10 Controls: 10	4/6 1/6	Optovue	4.5 × 4.5	RPC whole enface VD, RPC inside disc VD, RPC peripapillary VD
Aghsaei Fard et al. [24]	Iran	55.46 ± 11.38 55.26 ± 15.69	Cross-sectional study	Cases: 37 Controls: 54	21/16 22/32	Optovue	4.5 × 4.5	RPC whole enface VD, p-RNFL thickness, macular GCC thickness
Al-Nashar and Hemeda [25]	Egypt	60.2 ± 3.5 60.2 ± 3.5	Cross-sectional study	Cases: 25 Controls: 25	14/11 14/11	Optovue	4.5 × 4.5	RPC whole enface VD, RPC inside disc VD
Augstburger et al. [26]	France	66.9 ± 10.1 66.0 ± 9.2	Retrospective case-control study	Cases: 26 Controls: 24	16/8 16/8	Optovue	3 × 3	FAZ-S, FAZ-D, FSVD, RPC whole enface VD, RPC peripapillary VD, p-RNFL thickness, macular GCC thickness
Fard et al.[27]	USA	54.1 ± 11 58.4 ± 10.3	Cross-sectional study	Cases: 31 Controls: 77	16/15 35/42	Optovue	4.5 × 4.5	RPC whole enface VD, p-RNFL thickness
Fard et al. [28]	Iran	55.2 ± 11.8 47.0 ± 11.1	Cross-sectional study	Cases: 29 Controls: 48	14/15 21/27	Optovue	4.5 × 4.5	RPC whole enface VD, p-RNFL thickness, macular GCC thickness
Hata et al. [29]	Japan	66.4 ± 14.2 61.3 ± 19.1	Prospective observational study	Cases: 15 Controls: 19	8/3 6/8	Optovue	3 × 3	RPC whole enface VD, p-RNFL thickness
Liu et al. [30]	Taiwan	59.0 ± 10.7 51.4 ± 14.1	Prospective observational study	Cases: 13 Controls: 18	6/7 7/11	Optovue	4.5 × 4.5	RPC whole enface VD, RPC peripapillary VD, p-RNFL thickness, macular GCC thickness
Liu et al. [31]	Taiwan	59.90 ± 10.70 55.74 ± 14.51	Cross-sectional study	Cases: 10 Controls: 27	4/6 14/13	Optovue	4.5 × 4.5	RPC whole enface VD, RPC peripapillary VD, p-RNFL thickness, macular GCC thickness
Pierro et al. [32]	Italy	46.9 ± 12.5 47.5 ± 8.3	Cross-sectional study	Cases: 15 Controls: 15	9/6 8/7	Topcon	4.5 × 4.5	RPC whole enface VD, p-RNFL thickness
Sharma et al. [33]	Singapore	69 (61-82) 68 (52-82)	Observational case-control study	Cases: 6 Controls: 19	3/2 None	Optovue	4.5 × 4.5	RPC whole enface VD, RPC inside disc VD
Song et al. [34]	China	56.4 ± 8.38 55.57 ± 9.28	Cross-sectional study	Cases: 41 Controls: 30	14/16 12/18	Optovue	4.5 × 4.5	RPC whole enface VD, RPC inside disc VD, RPC inside disc VD

**Table 2:** NOS for assessing study quality.

Study	Selection	Comparability	Exposure	Total score
Fard et al. [19]	3	2	3	8
Mastropasqua et al. [20]	3	2	3	8
Abri Aghdam et al. [23]	3	0	3	6
Aghsaei Fard et al. [24]	3	2	3	8
Al-Nashar and Hemeda [25]	3	2	3	8
Augstburger et al. [26]	3	1	3	7
Fard et al. [27]	3	2	3	8
Fard et al. [28]	3	2	3	8
Hata et al. [29]	3	2	3	8
Liu et al. [30]	3	2	3	8
Liu et al. [31]	3	2	3	8
Pierro et al. [32]	2	2	3	7
Sharma et al. [33]	3	2	3	8
Song et al. [34]	3	2	3	8

## Data Availability

All data are fully available without any restrictions.
